# Hallmarks of Severe COVID-19 Pathogenesis: *A Pas de Deux* Between Viral and Host Factors

**DOI:** 10.3389/fimmu.2022.912336

**Published:** 2022-06-10

**Authors:** Roberta Rovito, Matteo Augello, Assaf Ben-Haim, Valeria Bono, Antonella d’Arminio Monforte, Giulia Marchetti

**Affiliations:** Clinic of Infectious Diseases and Tropical Medicine, Azienda Socio Sanitaria Territoriale (ASST) Santi Paolo e Carlo, Department of Health Sciences, University of Milan, Milan, Italy

**Keywords:** SARS-CoV-2, COVID-19, severity, immune dysregulation, biomarker, immunity, gut-lung axis, microbiota

## Abstract

Two years into Coronavirus Disease 2019 (COVID-19) pandemic, a comprehensive characterization of the pathogenesis of severe and critical forms of COVID-19 is still missing. While a deep dysregulation of both the magnitude and functionality of innate and adaptive immune responses have been described in severe COVID-19, the mechanisms underlying such dysregulations are still a matter of scientific debate, in turn hampering the identification of new therapies and of subgroups of patients that would most benefit from individual clinical interventions. Here we review the current understanding of viral and host factors that contribute to immune dysregulation associated with COVID-19 severity in the attempt to unfold and broaden the comprehension of COVID-19 pathogenesis and to define correlates of protection to further inform strategies of targeted therapeutic interventions.

## 1 Introduction

Since the end of 2019, the Severe Acute Respiratory Syndrome Coronavirus 2 (SARS-CoV-2, previously referred to as 2019-nCoV) was identified as the etiological agent of Coronavirus Disease 2019 (COVID-19) (1). SARS-CoV-2 is an enveloped virus, positive-sense single-stranded RNA, of the Coronaviridae family (2), the third of zoonotic transmission causing a large outbreak in humans. Indeed, SARS-CoV emerged in South China in 2002 (3), the Middle Eastern respiratory syndrome coronavirus (MERS-CoV) in Saudi Arabia in 2012 (4), and SARS-CoV-2 in the Chinese province of Hubei in 2019. COVID-19 is characterized by a broad spectrum of clinical manifestations, ranging from flu-like symptoms to systemic inflammation and multi-organ failure (5–10). Severe COVID-19 occurs in 14% of infected individuals, whereas critical COVID-19 in 5% (11).

SARS-CoV-2 is primarily transmitted by a direct, person-to-person transmission *via* the respiratory route, whereby the virus is suspended either on droplets or, less commonly, on aerosols (12). SARS-CoV-2 primarily infects epithelial cells in the airways *via* angiotensin-converting enzyme 2 (ACE2), its primary target receptor. The binding is followed by a proteolytic activation at the plasma membrane by the transmembrane protease serine 2 (TMPRSS2) or at the endosomal membrane by cathepsin L (13). The infection of the lower respiratory tract is followed by apoptosis, as a consequence of the viral replicative cycle, and by recognition of the viral infection by tissue-resident immune cells, as well as a first wave of inflammation due to macrophage and neutrophils activation that attempt to control the infection. This is followed by the recruitment of other immune cells from the bloodstream, further exacerbating inflammation, and eventually leading to the cytokine storm (14, 15). This represents the basis of pulmonary pathology, characterized by alveolar damage, oedema, inflammatory infiltrates, and fibrin deposition, resulting in the development of bilateral interstitial pneumonia (16–18). Worsening of this clinical picture involves manifestation of acute respiratory distress syndrome (ARDS), vascular damage, thrombosis, disseminated intravascular coagulation (CID), and multi-organ damage (7, 19).

ACE2 is also widely expressed in many other tissues, including the oral and nasal mucosa, nasopharynx, stomach, small intestine, colon, skin, lymph nodes, thymus, bone marrow, spleen, liver, kidney, testis, and brain (20), as well as in the endothelial and smooth muscle cells of these organs. In agreement with these findings, several studies detected the presence of SARS-CoV-2 in multiple organs of COVID-19 patients, including the brain, intestine, pharynx, heart, liver, kidneys, testicles, and blood, demonstrating its multi-organ tropism (21–23). How the virus disseminates onto these organs is still not fully understood.

Since the beginning of the pandemic, many definitions of COVID-19 severity have been employed, each focusing on different clinical aspects of the disease or referring to diverse laboratory and instrumental cut-offs, therefore hindering the reliable comparison of different studies and, in turn, the recognition of specific factors associated with a poor prognosis (24–32). To date, the most used classification system is the one developed by the World Health Organization (WHO), which distinguishes between four degrees of disease severity, based on clinical indicators: mild, moderate, severe, and critical (33), as summarized in **Table 1**.

**Table 1:** WHO COVID-19 disease severity classification in adults.

Disease severity degree	Characteristics	Definitions
**Mild disease**	No pneumonia	Symptomatic patients meeting the case definition for COVID-19 without evidence of viral pneumonia or hypoxia.
**Moderate disease**	Pneumonia	Patients with clinical signs of pneumonia (fever, cough, dyspnea, fast breathing) but no signs of severe pneumonia, including SpO_2_ ≥ 90% on room air.
**Severe disease**	Severe pneumonia	Patients with clinical signs of pneumonia (fever, cough, dyspnea) plus one of: •respiratory rate > 30 breaths/min; •severe respiratory distress; •or SpO_2_ < 90% on room air. While the diagnosis can be made on clinical grounds; chest imaging (radiograph, CT scan, ultrasound) may assist in diagnosis and identify or exclude pulmonary complications
**Critical disease**	Acute respiratory Distress Syndrome (ARDS)	Onset: within 1 week of a known clinical insult (i.e. pneumonia) or new or worsening respiratory symptoms. Chest imaging (radiograph, CT scan, or lung ultrasound): bilateral opacities, not fully explained by volume overload, lobar or lung collapse, or nodules. Origin of pulmonary infiltrates: respiratory failure not fully explained by cardiac failure or fluid overload. Need objective assessment (e.g. echocardiography) to exclude hydrostatic cause of infiltrates/oedema if no risk factor present. Oxygenation impairment: •Mild ARDS: 200 mmHg < PaO_2_/FiO_2_ ≤ 300 mmHg (with PEEP or CPAP ≥ 5 cmH2O); •Moderate ARDS: 100 mmHg < PaO_2_/FiO_2_ ≤ 200 mmHg (with PEEP ≥ 5 cmH2O); •Severe ARDS: PaO_2_/FiO_2_ ≤ 100 mmHg (with PEEP ≥ 5 cmH2O).
	Sepsis	Acute life-threatening organ dysfunction caused by a dysregulated host response to suspected or proven infection. Signs of organ dysfunction include: altered mental status (delirium), difficult or fast breathing, low oxygen saturation, reduced urine output, fast heart rate, weak pulse, cold extremities or low blood pressure, skin mottling, laboratory evidence of coagulopathy, thrombocytopenia, acidosis, high lactate, or hyperbilirubinemia.
	Septic shock	Persistent hypotension despite volume resuscitation, requiring vasopressors to maintain MAP ≥ 65 mmHg and serum lactate level > 2 mmol/L.
	Acute thrombosis	Acute venous thromboembolism (i.e. pulmonary embolism), acute coronary syndrome, acute stroke.

SpO_2_, oxygen saturation; CT, computed tomography; PaO_2_, arterial partial pressure of oxygen; FiO_2_, fraction of inspired oxygen; CPAP, continuous positive airway pressure; PEEP, positive end-expiratory pressure; MAP, mean arterial pressure.

Several clinical conditions have been associated with an increased risk of severe COVID-19, and include older age, chronic lung disease, cardiovascular disease, obesity, diabetes mellitus, liver disease, end-stage renal disease, and immunocompromise. However, the specific pathogenetic factors that dictate whether an individual will develop the severe form of the disease still remain a matter of scientific debate. The present narrative review article will shed light on our current understanding of the immune dysregulation associated to COVID-19 severity and the underlying viral and host pathogenetic mechanisms.

## 2 Immune Dysregulation in Severe COVID-19

**Figure 1:**
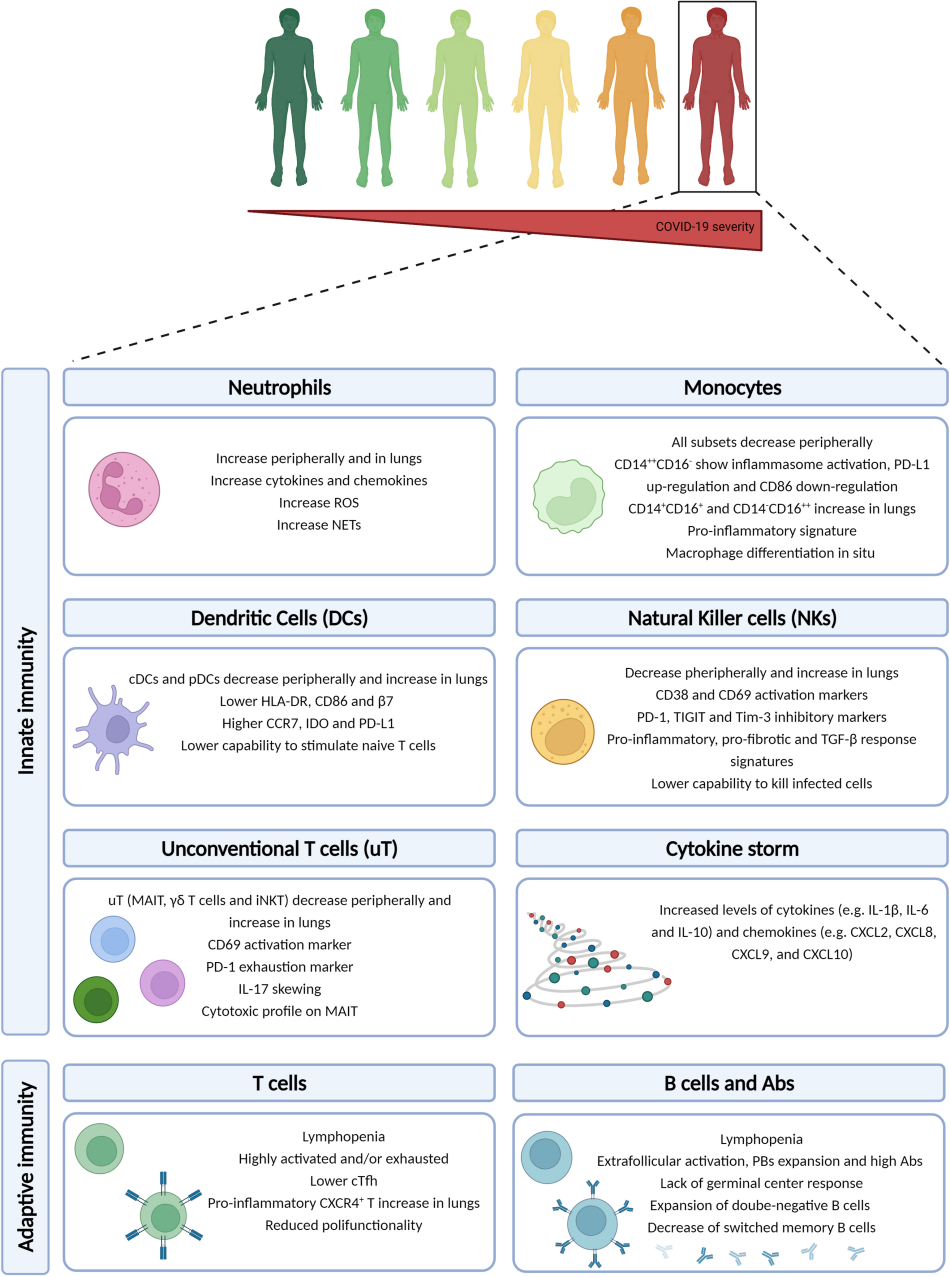
Immune dysregulation in COVID-19 severe patients. The severe/critical form of COVID-19 disease is associated to a multi-layered immune dysregulation involving both innate and adaptive immune responses. Created with BioRender.com.

The ultimate goal of the immune response is to clear the pathogen and develop a memory so that at the second encounter, the pathogen can be quickly cleared. The development of an antigen-specific memory immune response is a multifactorial process that begins with the initial viral detection by pathogen recognition receptors (PRRs), e.g. Toll-like receptors (TLRs), to initiate the IFN response. The initiation of the innate machinery may occur already a few hours post-infection and, while controlling the viral replication, tries to prime the adaptive immune response to produce cytotoxic and memory cells. The immune system of severe COVID-19 patients present a peculiar array of alterations compared to mild ones, which will be briefly described as follows (**Figure 1**).

### 2.1 Innate Immunity

#### 2.1.1 Cytokine Storm

An exaggerated inflammatory response is the main feature of severe and critical COVID-19 that was soon well documented by several reports (34–36). In general, serum levels of IL-2R, IL-4, IL-6, TNF-α, IL-1RA, IL-1β, IFN-γ are elevated (37), in conjunction with increased levels of chemokines such as CCL2, CCL8, CXCL2, CXCL8, CXCL9, and CXCL16 (38). Some of these cytokines (IL-6, IL-10) and chemokines (CXCL9, CXCL10) were found to be significantly higher in severe patients when compared to milder ones (39). IL-6 has a pivotal role in driving the hyperinflammatory response (35, 40, 41), and it is an independent predictor of patient survival (42). Increased CXCL2, CXCL8, IL-6, and IL-1β are consistent with neutrophilia that is often seen in COVID-19 patients, particularly in those who are severely ill (34, 36, 43, 44). The cytokine storm has been suggested to be characterized by a positive feedback loop. Indeed, the initial wave of cytokines may induce a form of inflammatory cell death (i.e. PANoptosis, composed of pyroptosis, apoptosis, and necrosis), that further induces the release of cytokines, eventually leading to the cytokine storm (45, 46). The PANoptosis seems to be associated to the concomitant increase of IFN-γ and TNF-α. A recent study showed that the change in cytokines and chemokines occurs within the first few days from symptoms onset (47), suggesting that the dysregulation may occur very early in the disease course. Finally, the overall increase of cytokines is usually followed by elevated clinical laboratory parameters such as C reactive protein (CRP), ferritin, and D-dimer, which are well-established acute phase proteins, as well as alanine aminotransferase (ALT), aspartate aminotransferase (AST), and lactate dehydrogenase (LDH), that are markers of tissue damage (43, 48).

Soon after the pandemic started, the cytokine storm became the hallmark of severe COVID-19. However, the cytokine storm is an important feature also in other respiratory infections. For instance, influenza virus infection, which can result in a broad range of clinical manifestations, is associated to a remarkable pro-inflammatory response that, in absence of suitable anti-inflammatory responses, leads to the establishment of the cytokine storm (49–52). Similarly to COVID-19, the cytokine storm mainly occurs in severe cases suggesting a common need to give more insights into the underlying mechanisms to guide future targeted therapeutic interventions.

#### 2.1.2 Neutrophils

Neutrophils are crucial early mediators against infections, as they can rapidly clear pathogens. In particular, neutrophils can employ various intracellular and extracellular mechanisms to control infections, i.e. phagocytosis, the release of cytokines and chemokines, production of reactive oxygen species (ROS), and release of neutrophil extracellular traps (NETs). NETs consist of an extracellular mixture of chromatin, microbicidal molecules, and oxidant enzymes in an attempt to control the infection. However, when these processes are dysregulated, the tissue damage due to hyperinflammation may take over. In the context of COVID-19, neutrophils’ amount and functionality have been associated to disease severity, with an increase at the local and systemic level (48, 53, 54). In particular, a substantial increase in neutrophils has been shown in severe compared to mild COVID-19 (55); besides, they had a more activated phenotype and were characterized by NETosis (54, 56). Indeed, in serum of COVID-19 patients various elements of NET have been identified, e.g. citrullinated histone H3 and myeloperoxidase (MPO) (57). Interestingly, when serum of COVID-19 patients is incubated with neutrophils isolated from healthy individuals *in vitro*, the release of DNA-bound MPO enzyme and extracellular chromatin structures with neutrophils elastase were observed, suggesting a remarkable NET trigger (57). The ability of NETs to exacerbate inflammation and microvascular thrombosis is in line with the pro-thrombotic events observed in severe COVID-19 patients. Finally, severe patients were characterized by occurrence of pro- and pre-neutrophils precursors, as well as a transcriptional status of dysfunctional and immunosuppressed neutrophils (58).

#### 2.1.3 Monocytes

The second major player during the first wave of innate response is represented by the monocyte subset. Monocytes are quickly recruited at the site of inflammation and can differentiate into macrophages or monocyte-derived dendritic cells (DCs) (59). Monocytes are further divided into immature classical (CD14^++^CD16^-^), differentiated inflammatory transitional (CD14^+^CD16^+^) and non-classical (CD14^-^CD16^++^) subsets.

In general, the peripheral blood of COVID-19 patients is characterized by a reduction of the various monocytes subsets, and such reduction increases with severity (60). Additionally, the classical CD14^++^CD16^-^ monocytes of severe COVID-19 patients showed inflammasome activation, as evidenced by caspase-1/ASC-speck activation (61). However, the down-regulation of the co-stimulatory CD86 with the concurrent up-regulation of the inhibitory PD-L1 molecule in classical monocytes coincided with a reduced capability to stimulate naïve T cells *in vitro* (62), suggesting that the enhanced pro-inflammatory status eventually may lead to dysfunction. Furthermore, the reduction of transitional and non-classical monocytes in peripheral blood is paired with selective recruitment in the lungs and with an increase of peripheral inflammatory markers such as IL-6 (60). The peripheral reduction and local increase of monocytes were more marked in those patients with superinfections in the lungs (60). Furthermore, CD16^hi^CD14^lo/-^ monocytes enriched in the lungs were characterized by expression of HLA-DR, suggesting non-classical monocyte-derived macrophages differentiation *in situ* (60), whereas the remaining circulating monocytes of severe patients are characterized by lower expression of HLA-DR (63, 64). Other studies have shown an abundant pool of pro-inflammatory monocyte-derived macrophages in the lungs (48), expressing CXCL10^+^CCL2^+^ with a pro-inflammatory transcriptomic signature composed of markers such as STAT1, IFNGR1, IFNGR2, NFKB1 and IL1B (65). These pro-inflammatory mediators can contribute to the immunopathology of the lungs.

#### 2.1.4 Dendritic Cells

Dendritic cells (DCs) consist of two main types, i.e. conventional or myeloid DCs (cDCs), i.e. CD1c^+^CD141^+^, and type I IFN-producing plasmacytoid DCs (pDCs), i.e. CD123^hi^. Within cDCs other subsets may be distinguishable, aimed at cross-presenting antigens to CD8 T cells (cDC1), initiating Th response (cDC2), or sharing features of cDC2 and monocytes (DC3) (62, 66). Therefore, DCs may have a pivotal role in the response against SARS-CoV-2 infection as they act at the bridge between innate and adaptive response.

Overall, SARS-CoV-2 infection induces a peripheral reduction of both subsets (67, 68), with a parallel influx in lungs and lymph nodes (48, 68). The immunophenotype of the DCs subset with regard to the homing and activation patterns were also altered. In particular, a lower percentage of the homing receptor integrin-β7+ (β7) and CD86+ DCs, as well as a higher percentage of the C-C chemokine receptor type 7+ (CCR7) and of the marker of immune tolerance and suppression indoleamine 2,3-dyoxigenase (IDO) were observed in COVID-19 patients. Importantly, the alterations initially observed have been shown to persist up to 7 months from infection, regardless of hospitalization (68). The overall reduction of DCs observed during SARS-CoV-2 infection is more pronounced in severe COVID-19 patients and refers to both populations, cDCs, and pDCs (60, 64, 67, 69), regardless of whether they presented superinfections in the lungs (60). Severe COVID-19 patients presented selective recruitment in lungs in particular of cDC2 (60). Additionally, within the DC3 subset, an increase of CD163^+^CD14^+^ was observed in severe COVID-19 (62).

Finally, the reduction of DCs in the peripheral blood of COVID-19 patients is accompanied by altered functionality (64, 67, 69). For example, cDCs and pDCs are impaired in their capacity of producing cytokines in response to TLR stimulation (64), and in their capability to stimulate naïve T cells *in vitro*, thus priming the adaptive immune responses, which correlated with disease severity (62). Such impairment coincided with lower levels of HLA-DR, CD86, and increased levels of the inhibitory PD-L1 molecule (62, 64, 67, 69).

#### 2.1.5 Natural Killer Cells

Natural killer (NK) cells are divided into cytokine-producing (i.e. CD56^bright^) and cytotoxic (i.e. CD56^dim^) NK cells. Despite NK cells have mainly antiviral and antitumoral functions, they can also control fibrogenesis.

Overall, a feature of COVID-19 is the reduction of NK cells at the periphery with a parallel increase in the lungs (48, 70, 71), with an expression of both activation markers, such as CD38 and CD69 (70, 72), and inhibitory receptors, such as NKG2A, PD-1, CD39, TIGIT, and Tim-3 (70, 71, 73), particularly in severe patients. Importantly, NK cells of severe COVID-19 patients showed a distinct gene expression signature compared to those with a milder form of disease, e.g. an IFN-α signature (71). Furthermore, in parallel with such re-distribution, the immunophenotype was altered, with enrichment of inflammatory and proliferating cytotoxic CD56^dim^ NK cells (71), as well as adaptive CD57^+^ NK cells expressing high levels of perforin and NKG2C (70, 74).

Additionally, with re-distribution and immunophenotype alteration comes functional impairment. Indeed, normal NK cells from healthy donors were able to reduce viral proteins when co-cultured with SARS-CoV-2-infected lung fibroblasts *in vitro*, as well as to reduce the expression of pro-fibrotic genes such as COL1A and ACTA2 (71). Whereas, NK cells from severe COVID-19 patients were not able to reduce viral proteins nor to suppress pro-fibrotic genes (71). Indeed, NK cells of severe COVID-19 patients showed an expression signature that is typical of pulmonary NK cells of patients with lung fibrosis (71). Similar findings were shown in another study of NK cells co-culture with SARS-CoV-2 infected cells *in vitro*, from both healthy and COVID-19 donors: NK cells from healthy donors killed SARS-CoV-2 infected cells in a dose-dependent manner, whereas this did not occur in NK cells from severe COVID-19 patients, in whom lower NK cells counts also associated to a slower decline of viral load (75). Interestingly, TGF-β was identified as having a putative role in such impairment as NK cells showed a remarkable TGF-β response signature, characterized by downregulation of T-bet and integrin-β2, that prevent the proper NK cells binding to SARS-CoV-2 infected cells, granule exocytosis and cell-mediated cytotoxicity (75). Interestingly, incubation of NK cells from healthy individuals with plasma of severe COVID-19 patients *in vitro* induced impairments in NK cells, which is in line with the positive correlation between reduction and impairment of NK cells with the systemic hyperinflammation (71, 75). Therefore, hyperactivation and hyperinflammation are important mechanisms of NK cells impairment, as described in the settings of chronic stimulation (76).

### 2.2 Unconventional T Cells

Unconventional T (uT) cells represent about 10% of circulating T cells and are a major component of the mucosal immune system, exhibiting both innate and adaptive features. These immune cells express invariant TCRs that recognize nonpeptide antigens in an MHC-unrestricted manner and can promptly respond upon activation with the production of cytokines, e.g. IFN-γ, TNF-α, and IL-17A, and cytotoxic activity without undergoing clonal expansion and differentiation into effector cells. This heterogeneous population encompasses three main lineages, i.e., mucosa-associated invariant T (MAIT), γδT, and invariant natural killer T (iNKT) cells, which are engaged in mucosal homeostasis as well as in antitumor and antimicrobial immunity (77).

MAIT cells, Vδ2+ γδT cells as well as iNKT cells appear to be greatly depleted in the peripheral blood of COVID-19 patients in a severity-dependent manner (78–83). Residual circulating uT cells show a significant increase in the expression of both activation (78, 80, 81, 84) and exhaustion (78) markers, i.e. CD69 and PD-1, respectively. CD69 expression on MAIT cells has shown to be more pronounced in patients with detectable SARS-CoV-2 viremia (80), as well as in those with a more severe disease (80, 81, 84), suggesting a striking association between MAIT cells activation and worse clinical outcome. On the contrary, another study found a positive correlation between CD69 expression on MAIT and iNKT cells at the time of admission and the PaO_2_/FiO_2_ ratio, suggesting a possible beneficial role of early MAIT and iNKT cells activation in severe COVID-19 (78). Circulating uT cells of COVID-19 patients produce less IFN-γ in the backdrop of more IL-17A, a pro-inflammatory and pro-fibrotic cytokine (78), whereas MAIT cells additionally showed an increased Granzyme B production, with a direct correlation between MAIT cytotoxic activity and severity of disease (80, 81).

The reduction of circulating uT cells is paralleled by increased proportions of MAIT (78, 80) and γδT cells (85), expressing high levels of activation and exhaustion markers in endotracheal aspirates, bronchoalveolar fluid, and pleural effusions of COVID-19 patients, suggesting recruitment of these cells in inflamed lungs. This is further supported by a high expression of MAIT cells chemoattractant such as CXCL10 in airway fluids (78), as well as a reduced expression of chemokine receptors involved in lung homing, e.g. CXCR3 or CCR6, on circulating MAIT cells. Finally, as shown for circulating MAIT cells, also local cells show increased production of IL-17A and Granzyme B in the backdrop of decreased IFN-γ (80, 81). The pro-inflammatory IL-18 produced by monocyte/macrophages has been described as one of the main driving factors for uT cells activation and cytotoxicity. Indeed, a positive correlation between plasmatic IL-18 and CD69 on MAIT and iNKT cells, as well as Granzyme B by MAIT cells, was described (78, 81).

Taken together, these data suggest recruitment of uT cells to the lungs as well as an excessive response with heightened cytotoxicity and skewing toward IL-17A production, which can contribute to lungs inflammation and fibrosis. Interestingly, the similarities of MAIT cells alterations between COVID-19 and obese patients (86–88), could partially explain the poorer COVID-19 outcome in people with obesity (89).

### 2.3 Adaptive Immunity

The clinical aggravation of COVID-19 occurs approximately one week from symptoms onset, which corresponds to the temporal bridging between innate and adaptive immunity (7, 9, 90), suggesting dysfunction in one or more steps of the adaptive immunity priming. Lymphopenia is a common finding amongst patients with SARS-CoV-2 infection that correlates with increased severity (10, 34, 36, 91). Pathological studies have also documented lymphocytes depletion in secondary lymphoid organs specimens (92, 93). Therefore, the use of lymphopenia and neutrophilia as a combined marker (neutrophil to lymphocyte ratio, NLR) has been suggested crucial in assessing disease severity (94, 95).

#### 2.3.1 Immunophenotypes

Deep immune profile analysis of hospitalized COVID-19 patients showed a highly heterogeneous adaptive response and identified two main distinct immunophenotypes: immunophenotype 1, associated with severity, characterized by highly activated CD4 T cells, fewer circulating T follicular helper (cTfh) cells, and hyperactivated or exhausted CD8 T cells; immunophenotype 2, associated with a more favorable outcome, including less activated CD4 T cells, Tbet^+^ effector CD4 and CD8 T cells, and proliferating B cells (96). The activated features of phenotype 1 have been described in several reports (39, 63), as well as the exhaustion features (36, 41, 48, 97–101). Exhaustion is a multifactorial and multifaceted state characterized by expression of various inhibitory receptors (e.g. PD-1, Tim-3, CTLA-4, and TIGIT), as well as low proliferative capacity, reduced cytokines production and cytotoxic potential of immune cells. It is plausible that immune exhaustion in COVID-19 is a consequence of the general prolonged activation and inflammation. Furthermore, CD4 T cells differentiation into various subsets, e.g. Th1 and Th2, has been shown to have an important role in influencing the clinical outcome of infections such as HIV (102). In the context of COVID-19, despite the general lymphopenia, an incorrect Th polarization has been shown to be associated with outcome in several studies (103–105). In particular, a polarization toward Th2 was associated with disease severity and with a worse outcome. This may be due to the intrinsic features of such subsets. Indeed, the Th1 subset coordinates the antiviral response by means of CTLs and NKs activation; whereas the Th2 subset activates cells such as eosinophils and basophils, thus tends to inhibit the Th1 response.

#### 2.3.2 Antigen-Specific T Cell Responses

The antigen-specific T cell response is highly heterogeneous, both in the CD4 and CD8 T cells subsets. Indeed, virus-specific T cells can differentiate into a broad range of subpopulations with different effector functions such as: direct antiviral activity *via* cytokines secretion and recruitment of many immune cells (i.e. T helper cells, Th); cytotoxicity (i.e. CD8 T cells); support of B cells maturation and antibody production (i.e. T follicular helper cells, Tfh). The goal of the adaptive immune response is to clear the infection and build a memory to rapidly intervene upon secondary encounter with the pathogen. Memory T cells differentiate from naïve T cells into central memory T cells (T_CM_), effector memory T cells (T_EM_), and terminally differentiated effector memory T cells (T_EMRA_).

In response to SARS-CoV-2 infection, antigen-specific T cells are generally produced (106), as early as two days post-symptoms onset, with CD4 T cells frequencies overcoming those of CD8 T cells (107, 108). The presence of SARS-CoV-2-specific CD4 and CD8 T cells has been associated with milder disease (106, 107, 109). Furthermore, the functional properties were altered in severe COVID-19 patients, as showed by a lower percentage of polyfunctional CD4 T cells producing simultaneously the classical Th1 cytokines, i.e. IFN-γ, TNF-α, and IL-2 (110). The importance of SARS-CoV-2-specific T cells is further supported by the infection resolution in a COVID-19 patient who did not produce neutralizing antibodies but had instead antigen-specific CD4 and CD8 T cells (107). Interestingly, in critical/deceased COVID-19 patients, it has been shown that SARS-CoV-2-specific T cell responses occur and are comparable to that of moderate patients (111), though not able to clear the infection and/or limit the systemic damage.

SARS-CoV-2-specific T cells can migrate into the lungs as part of COVID-19 pathogenesis. An increased proportion of inflammatory CXCR4^+^ CD4 and CD8 T cells in the lungs of severe COVID-19 patients was shown (109), whereas in the lungs of moderate patients, an increased proportion of resident memory T cells was observed (48). Additionally, in a study of scRNA-seq on nasopharyngeal and lung samples, a transcriptional signature characterized by strong interactions between epithelial cells and hyperactivated T cells was described, pointing to a potential direct contribution of hyperactivated immune cells on epithelial cells damage (53). These data suggest that inflammatory and hyperactivated T cells are involved in immunopathology.

Finally, contrasting data exist on regulatory T cells (Treg). Despite the generally low levels of SARS-CoV-2-specific T cells described in COVID-19 patients, some studies described an increase of Treg frequencies in severe COVID-19 patients (97, 109, 112), whereas others did not find such association (34).

#### 2.3.3 Antigen-Specific B Cell and Humoral Responses

Humoral response is characterized by a first phase of short-lived low-affinity antibody-secreting plasmablasts (PBs), followed by a second phase of long-lived plasma cells and high-affinity memory B cells formed upon the germinal center response to generate long-lived plasma cells and high-affinity memory B cells. Therefore, PBs produced during the acute phase tend to quickly disappear, but upon re-infection memory B cells can rapidly form IgG-secreting PBs.

Though the majority of studies focus on T cell and antibody responses, perturbations in the B cell compartment associated with COVID-19 severity have been described. Severe COVID-19 patients showed extrafollicular B cells activation with expansion of PBs that inevitably produce high titers of antibodies, similarly to what was reported in autoimmune diseases, which strongly correlates with the level of proinflammatory cytokines (96, 113). The lack of a positive correlation between the titer of neutralizing antibodies and disease severity has been shown by several reports (114, 115), though others have found reduced levels of SARS-CoV-2-specific peripheral IgA, IgG, and IgG1 in severe/critical COVID-19 patients (116). In particular, such populations are characterized by CD19^+^CD27^+^CD38^hi^ antibody-producing cells, as well as CD11^+^ activated naïve B cells that differentiate in effector B cells lacking naïve (IgD) and memory (CD27) markers, i.e. CD19^+^CD27^-^CD38^-^CD24^-^IgD^-^CD11c^+^CD21^-^, also called double-negative B cells (113, 117). The expansion of B cells lacking memory markers has been described in several studies and has been referred to as atypical memory B cells (CD21^lo^CD27^-^CD10^-^) (118). Additionally, a decrease of switched memory B cells was suggested as independent risk factor for mortality in COVID-19 patients (116), whereas proliferating memory B cells were not associated with severity (96). Importantly, the antibody-secreting cells produce high levels of antibodies that can have neutralizing features in severe COVID-19 patients, as shown in several studies (63, 119, 120). However, many severe COVID-19 patients fail to develop the germinal center reaction, as shown in studies of deceased COVID-19 patients where a lack of germinal centers response in spleen and lymph nodes, as well as a reduction of Tfh cells, was demonstrated (121). Indeed, this is further supported by the association of cTfh frequency with a reduced severity (107). Importantly, COVID-19 patients with somatic mutations in memory B cells presented a quick recovery from SARS-CoV-2 infection (122, 123).

The mechanisms underlying the lack of disease control in the backdrop of high levels of neutralizing antibodies still need further clarification, however, the aforementioned data suggest that an accurate B cell maturation, e.g. involving somatic hypermutation, is pivotal for the development of cells with high-affinity and breadth.

Finally, coordinated immune responses made by the three arms of the adaptive response, i.e. CD4 T cells, CD8 T cells, and antibodies, were shown to be associated with milder disease, whereas uncoordinated responses were more often seen in aged and severely ill individuals (107).

## 3 Underlying Mechanisms of Immune Dysregulation

**Figure 2:**
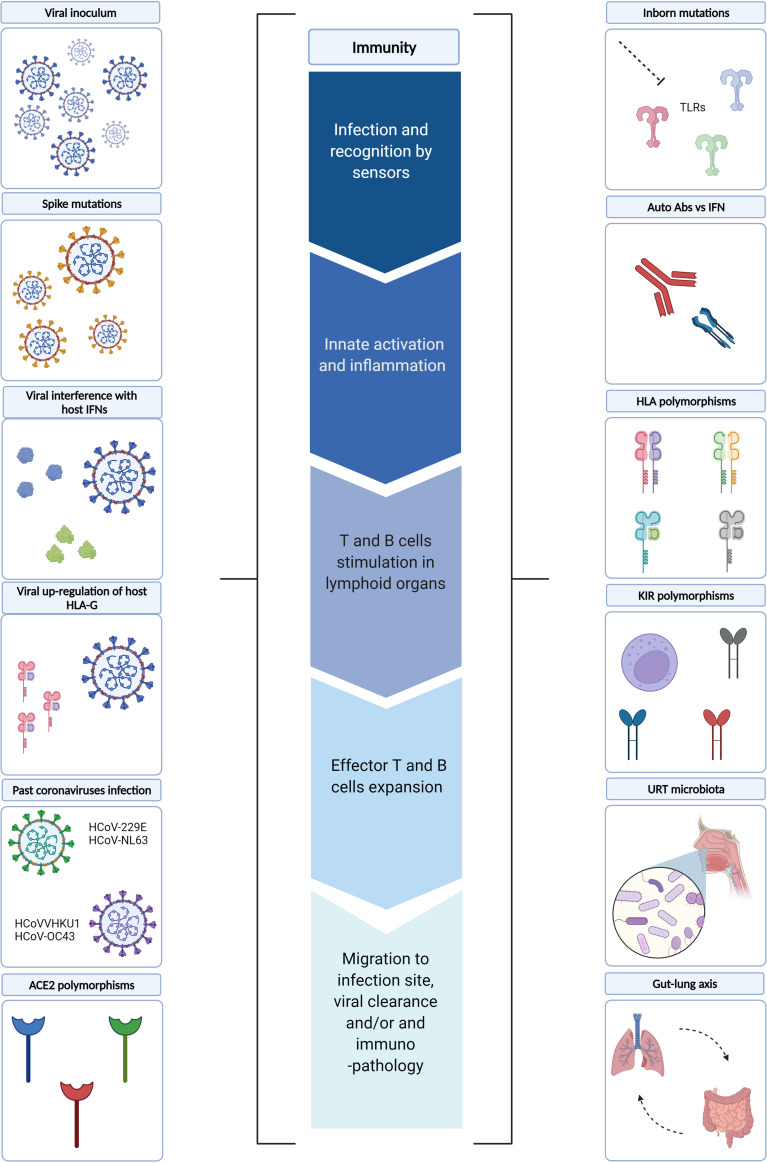
Underlying mechanisms of immune dysregulation. Several viral and host factors have been described as influencing one or more steps of the immune response during SARS-CoV-2 infection. In particular, the early phases of the immune response may be influenced by i) viral factors, such as viral inoculum, Spike mutations and viral interference of host IFN pathways, as well as ii) host factors, such as ACE2 polymorphisms and URT microbiota. Whereas viral up-regulation of host HLA-G, inborn host mutations, auto-reactive Abs *vs* IFN, HLA and KIR polymorphisms, past coronavirus infections may influence the later phases of immune responses. In this context, gut-lung axis perturbations may further fuel systemic inflammation. TLRs, Toll-like receptors; IFN, Interferon; Abs: Antibodies; HLA, Human Leukocyte Antigens; KIRs, Killer Cell Immunoglobulin-like Receptors; URT, Upper Respiratory Tract; ACE2, Angiotensin-converting Enzyme 2. Created with BioRender.com.

In the following sections, we will review the mechanisms underlying the immune dysregulation described in severe COVID-19 patients. In particular, viral and host factors, as well as the role of microbiota (**Figure 2**).

### 3.1 Viral Factors

The SARS-CoV-2 viral load an individual is infected with, i.e. viral inoculum, certainly represent a first viral factor that may potentially shape the immune responses. Given the severity of the disease, human challenge studies aimed at evaluating disease severity in relation to the initial viral inoculum are lacking; however, several animal studies suggest interesting associations. In a murine model of mouse-adapted SARS-CoV-2 intranasal infection, a dose-dependent increase in morbidity and mortality was observed (124). Furthermore, in a Syrian Hamster model of SARS-CoV-2 infection, the severity of pneumonia increased with viral inoculum when administered intranasally, whereas the oral administration yielded mild pneumonia (125). The use of animal models allows to exclude all variables that may influence the outcome, e.g. genetic background, immunity, and environment. In real-life settings, this cannot occur, and the studies evaluating SARS-CoV-2 viral load in relation to COVID-19 outcome quantify such parameter at hospitalization and/or at symptoms onset, i.e. several days from priming. Therefore, several mechanisms attempting to control a high viral inoculum may have already taken place. This may explain the apparently contrasting data among studies evaluating the association of viral load in nasopharyngeal swabs with COVID-19 outcome. Indeed, some reports have found an association with disease severity (24–28), whereas others did not find a strong association (29, 30), or did not even find it (31, 32).

Importantly, SARS-CoV-2 genome, proteins, or virus-like particles have been detected in many other compartments than the respiratory tract (21–23, 126, 127). How the virus spreads in the other organs is not fully understood, even though there is some evidence that the viral presence in the bloodstream, i.e. referred to as SARS-CoV-2 RNAemia, may be a first critical step. Whether the RNAemia corresponds to infectious viral particles is still under discussion. A study showed a direct cytopathic effect upon Vero-E6 cells infection with the plasma of an immunocompromised patient, and these cells produced virions (127). In the same study, infectious SARS-CoV-2 virus has been isolated from several tissues, i.e. heart and kidney (127). Another study visualized SARS-CoV-2 virions in centrifuged plasma pellets by means of several imaging-based approaches in a cohort of SARS-CoV-2 infected individuals with different degrees of severity, none of them were reported to be immunocompromised (128). Therefore, these data suggest that SARS-CoV-2 RNAemia could be partially explained by SARS-CoV-2 viremia. Despite the viral control in immunocompromised patients is highly impaired compared to immunocompetent individuals, similar mechanisms of replication-competent SARS-CoV-2 spreading may occur in those immunocompetent patients with multi-organ involvement. The detection of SARS-CoV-2 genome in the bloodstream occurs in a relatively small fraction of infected individuals (7, 24, 129, 130); its levels were consistently associated with poor outcomes and were mainly observed within critically ill patients (127, 128, 130–132). SARS-CoV-2 viral load measured in plasma is usually lower than the load measured in the respiratory tract and can be detected as early as the first week from disease onset (130, 133, 134), suggesting that in some patients can be an early event in the pathogenesis. The low levels of plasmatic SARS-CoV-2 may be a strong limiting factor to the isolation of sufficient replication-competent viruses. Interestingly, whereas a strong positive correlation is observed in SARS-CoV-2 viral load measured in different districts of the respiratory tract, their correlation with RNAemia significantly drops (130, 135). However, those individuals with high viral loads both in the lungs and in the plasma succumb rapidly (127). These different mechanisms may underly compartmentalized immune responses (135), and potential organ-specific evolution, as shown in immunocompromised patients (127).

Finally, SARS-CoV-2, as any other RNA virus, is characterized by high mutation and recombination rates (136), with heterogeneous consequences in the viral proteins. Each mutation may confer a new/improved skill that, together with those of the other mutations, confer at the new strain the typical characteristics of a Variant of Concern (VOC) that negatively impacts public health. To date, five VOCs have been identified by the World Health Organization (WHO), i.e. B.1.1.7 (Alpha), B.1.351 (Beta), P.1 (Gamma), B.1.617.2 (Delta), and B.1.1.529 (Omicron). Some of the accumulated mutations possess important immune evasion features that could influence the immune dysregulation observed in COVID-19 patients, e.g. the mutation E484K in the RBD portion of the Spike protein, present in the Beta and Gamma VOCs. In *in vitro* and in deep mutational scanning (DMS) studies E484K has shown to induce evasion both from monoclonal treatment and humoral response (137, 138). Additionally, the most recent Omicron VOC, which is heavily mutated, with 15 out of 30 mutations located in the RBD portion, contains various mutations that could individually escape neutralizing Abs, i.e. K417N, G446S, E484A, and Q493R (139). This may occur in the backdrop of mutations that confer an advantage on transmissibility, e.g. the mutation N501Y, present in Alpha, Beta, and Gamma VOCs, which increases the affinity of the RBD for ACE2, thus increasing transmissibility (140). It has also been shown both *in vitro* and in animal models that such mutation increases the rate of virus replication (141). In the Omicron VOC, the additional Q498R mutation makes the binding affinity to ACE2 even stronger (142). The Spike mutation E484K present in the Beta and Gamma VOCs also increases the affinity of the RBD for ACE2 (143). Additionally, the mutation P618H, present in Alpha and Omicron, can greatly enhance spike cleavage, thus increasing transmission (142). Interestingly, a study of replication competency in human *ex vivo* explant cultures has shown that Omicron replicates faster than all the other VOCs in the bronchus but less efficiently in the lung, which can partially explain the reduced severity in the backdrop of an enhanced transmissibility (144).

Viruses have developed countless countermeasures to directly influence the diverse aspects of human antiviral responses. The SARS-CoV-2 attempt to interfere with immunity starts with the innate response, which is the first line of defense aimed at containing viral replication and dissemination while priming the adaptive immune response. Several SARS-CoV-2 proteins have been described as affecting the IFN response. However, the most characterized so far are the non-structural protein 1 (nsp1) and ORF6, both of which have already been described as virulence factors in SARS-CoV infection (145, 146). In SARS-CoV-2 infection, they act *via* different mechanisms. Nsp1 reduces translation by means of the interaction with the 40S subunit of the ribosome, leading to lower interferons (IFNs) and interferon-stimulated genes (ISGs) products synthesis while protecting the viral translation (147, 148). ORF6 blocks the IRF3 and STAT1/2 nuclear translocation by means of interaction with export factors such as Rae1 and Nup98 (149, 150). Interestingly, a study evaluating the gene expression profile of several cell lines upon infection with different SARS-CoV-2 multiplicities of infection (MOIs), showed that low-MOI infection yielded a classical signature of reduced IFNs in the backdrop of increased chemokines, whereas high-MOI infection maintained high levels of IFN (38). These data may suggest that the action of viral antagonists of interferon signalling may be ineffective in conditions of high-MOI, as a high viral burden may be able to activate the usual innate antiviral mechanisms. Whether this occurs *in vivo* remains to be determined. Furthermore, as mentioned earlier, one of the hallmarks of severe COVID-19, as well as of severe influenza virus infection, is the establishment of the cytokine storm. Previously, much attention was put on the highly pathogenetic avian influenza virus H5N1 due to the zoonotic transmission and high mortality rate. Interestingly, the avian H5N1 virus could replicate more in myeloid cells compared to the human adapted virus, and the higher RNAs induced a striking cytokine production thus leading to a more severe outcome in murine models (151). This was attributed to internal viral genes such as polymerase and the non-structural proteins.

One of the mechanisms by which NK cells are activated is the “missing-self” mode by which infected cells down-regulate self-antigens. In particular, activating NK receptors recognize pathogens-induced markers, whereas inhibiting NK receptors bind to self-antigens such as the non-classical HLA class I molecule HLA-E. It has been shown that SARS-CoV-2 encodes a peptide, i.e. non-structural protein 13 (Nsp13), that binds to HLA-E stabilizing the complex on the surface of infected cells. However, instead of binding the inhibitory NKG2A receptor, it prevents such binding, thus leading to the activation of NK cells (152). However, whether it represents an advantage for the pathogen in viral infections such as CMV, characterized by an expansion of adaptive NKG2C+ NK cells, still remains to be elucidated (153).

An additional evasion mechanism employed by several viruses is the up-regulation of HLA-G in the host cell. HLA-G is a non-classical HLA Class I antigen that has immune inhibitory features *via* receptor signalling and exists in various forms, i.e. membrane-bound (HLA-G 1-4) and soluble (sHLA-G 5-7). Several polymorphisms have been described, some of which are associated with a higher expression (154), therefore, with a potential heightened inhibitory capacity. Upon SARS-CoV infection of lung epithelial Calu-3 cells, an upregulation of HLA-G was observed, whereas this did not occur when infecting with MERS-CoV (155). Unfortunately, very few reports exist on its potential clinical impact on SARS-CoV-2 infection and it certainly merits further evaluation. sHLA-G was found increased in severe COVID-19 patients compared to controls (156, 157); however, when followed up for a short period of time, an increase of sHLA-G was associated with a better outcome, most likely reducing the neutrophils adhesion (156). Moreover, in a study following the dynamic of HLA-G expression on various immune cells in a COVID-19 patient up to the convalescence phase, a high-low-high pattern of HLA-G was observed, most likely reflecting the SARS-CoV-2 infection dynamics (158).

### 3.2 Host Factors

#### 3.2.1 Smoke

Interestingly, a pre-existing pulmonary damage may play a role in COVID-19 severity. One of the most common habits that can underly pulmonary damage is smoke. Indeed, a worse outcome has been observed in smoking individuals (159). Smoke may also contribute to disease severity by impairing local immune responses and epithelial regenerative capacity. It has been shown *in vitro* that acute exposure of airway epithelium to cigarette smoke allows for more severe proximal SARS-CoV-2 induced airway epithelial damage by reducing the mucosal innate immune responses and the proliferation of airways basal stem cells (160).

#### 3.2.2 Sex and Age

Finally, soon after the pandemic started, sex and age were the first risk factors to be identified, i.e. worse outcomes in males and in elderly (161–163). These factors could be partially ascribed to intrinsic immune features. First of all, it is known that, overall, the immune responses elicited by females are somewhat enhanced than those elicited by males (164). Interestingly, in a murine model of SARS-CoV infection, estrogens were identified as a receptor signalling key for protection in females (165). Additionally, there might be genetic factors more frequently observed in males. For example, 90% of severe COVID-19 patients with type I IFN autoantibodies were reported to be male (166). Furthermore, COVID-19 has been inversely correlated with the frequency of naïve T cells (96, 107), and the pool of naïve T cells remarkably declines over time (167). The decline of naïve T cells observed with age is more pronounced in males compared to females and is associated to a male-specific decline in B-cell specific loci (168).

#### 3.2.3 Host Genetic Factors

A number of host genetic factors may differentially shape the initial viral binding, recognition, and immune activation. In particular, various polymorphisms of the ACE2 gene have been associated with disease severity as these may change the binding affinity to the Spike protein of SARS-CoV-2 (169). Furthermore, individuals with autoantibodies against IFN-I (166, 170), or inborn mutations resulting in defective genes related to IFN-I, e.g. TLR3, TLR7, and IRF7 (171–173), presented a worse outcome. The importance of an early immune activation is supported by the counter-productive action of early corticosteroid treatment, as it reduces the viral-induced danger signals necessary to activate the immune system (174).

After the initial viral recognition, viral antigens need to be processed and presented to immune cells *via* the Major Histocompatibility (MHC) molecules, coded by the human leukocyte antigens (HLAs) genes. An *in-silico* analysis of viral peptide-MCH class I binding affinity highlighted two HLA alleles with potential implications for COVID-19 severity (175). In particular, HLA-B*46:01 presented the fewest predicted binding peptides for SARS-CoV-2, suggesting that individuals carrying such allele may be more likely to develop severity, as shown for SARS-CoV infection (176). Conversely, HLA-B*15:03 showed a high capacity to present conserved peptides, i.e. shared among other coronaviruses, suggesting a potential cross-protection. In an Italian cohort, the HLA-DRB1*15:01, HLA-DQB1*06:02, and HLA-B*27:07 alleles were associated with severe disease (177). Furthermore, NK cells express a variety of killer cell immunoglobulin-like receptors (KIR), which are highly polymorphic glycoproteins that induce inhibitory or activating downstream signals. Usually, upon recognition of the self-HLA molecules, inhibitory KIRs are involved in suppressing NK cells, whereas the down-regulation of the self-HLA, upon viral infections, induces activation of NK cells. In the context of SARS-CoV-2, the KIR AA genotype was associated to a severe COVID-19 disease, in particular, the KIR2DS4 was the most significantly associated with severity, followed by KIR3DL1. Interestingly, the AA genotype encodes for various inhibitory KIRs and only one activating KIR (178).

As mentioned earlier, IL-6 is a key cytokine involved in the cytokine storm, i.e. a hallmark of severe COVID-19. Polymorphisms of IL-6 gene account for differences in plasmatic cytokine levels: the GG genotype (-174G/C) has been previously associated with higher circulating IL-6 (179). Interestingly, IL-6 polymorphisms have been described to affect disease course and response to therapy in several viral infections: the low IL-6-producing CC genotype has been associated with more severe symptoms during respiratory syncytial virus (RSV) infection (180), whereas the high IL-6-producing genotypes, GG or GC, were associated with sustained virologic response in patients with HIV/HCV co-infection treated with pegylated interferon-α (181).

In the context of COVID-19, contrasting data exist on the topic: some studies did not show an association between the abovementioned IL-6 polymorphism with COVID-19 severity, whereas others did find an association (182–184). Interestingly, polymorphisms in other cytokines have been associated to COVID-19 severity. In particular, the AA TNF-α genotype is associated with a more severe COVID-19 disease (185). Furthermore, polymorphisms in other cytokines and cytokine receptors such as IL-17 and IL10RB have been suggested as having a potential influence on COVID-19 progression (186, 187). While the whole genetic background and the immune signature of different populations are likely to influence the role these polymorphisms have on COVID-19 outcome, it appears that carriers of cytokine/cytokine receptors high-producing genotypes may negatively impact COVID-19 severity most likely through the enhancement of the pro-inflammatory milieu. Whether the simultaneous presence of high-producing polymorphisms of different cytokines may have a synergistic effect on the cytokine storm in severe COVID-19 still remains to be determined.

#### 3.2.4 Vitamin D

To date, the role of Vitamin D on COVID-19 severity is still controversial. Indeed, some studies found an association between Vitamin D and COVID-19 severity, whereas others did not find it (188–191). However, there is no doubt that vitamin D exerts important functions in antiviral immunity, as shown in various viral models. For instance, in a murine model of influenza virus infection, the daily supplementation of a high dose of 25-hydroxyvitamin D_3_ reduced the production of pro-inflammatory cytokines and improved the outcome (192). Whereas in humans, a meta-analysis study showed that the daily or weekly administration of Vitamin D reduced the incidence of acute respiratory tract infections, and that the protective effect was stronger in those individuals with a baseline level of Vitamin D below 25nmol/L (193). For these reasons, despite the controversial data, the role of Vitamin D in COVID-19 severity and outcome is still under evaluation, mostly for its potential therapeutic effect. Overall, it appeared that Vitamin D supplementation started during SARS-CoV-2 infection has limited to no effect on the disease course, whereas when administered regularly for a longer period of time before infection it may have a positive effect (188, 194–196), most likely reflecting the kinetic of vitamin D, that requires some time to reach a functional concentration (197). New insights into the mechanisms underlying the abovementioned associations are necessary to finally understand the actual role of Vitamin D in the pathogenesis of COVID-19.

#### 3.2.5 Exosomes

During viral infections, extracellular vesicles of 30-100 nm are normally produced from a broad array of cells and, in certain cases, can present viral antigens to the host immune system. A study evaluating exosomes isolated from plasma of COVID-19 patients has shown that exosomes of mild patients (which were of B cell, DC, and monocyte/macrophages origin) contained a higher number of SARS-CoV-2 antigens and were able to induce CD4 T cell activation compared to exosomes isolated from severe COVID-19 patients, suggesting that exosomes can contribute to prime adaptive immune responses, thus reducing the severity of disease (198).

#### 3.2.6 Previous Infection With Seasonal Coronaviruses

Seasonal coronaviruses share a certain degree of homology with SARS-CoV-2 and are frequently found in the population. Therefore, one of the aspects long debated is whether past seasonal coronavirus infection may confer any degree of cross-protection during SARS-CoV-2 infection, thus leading to less severe disease. The etiologic agents of the seasonal common cold are either alphacoronaviruses, i.e. HCoV-229E and HCoV-NL63, or betacoronaviruses, i.e. HCoVHKU1 and HCoV-OC43; SARS-CoV, MERS-CoV, and SARS-CoV-2 belong to the betacoronavirus genera (199).

Interestingly, several SARS-CoV-2-specific T cell studies reported that 20-50% of individuals never exposed to SARS-CoV-2 had significant T cell reactivity, mainly CD4 T cells (106, 200, 201), and all authors speculated that this phenomenon may have been due to preexisting memory responses against human “common cold” coronaviruses (HCoVs). Subsequently, this phenomenon was experimentally confirmed in a study evaluating the antigen-specific CD4 T cells response after stimulation with HCoV epitopes homologous to SARS-CoV-2 in unexposed donors and SARS-CoV-2 convalescent (202). The same trend was observed for memory B cells and humoral response. In particular, in a study comparing serum antibodies and memory B cell responses to coronavirus spike proteins in pre-pandemic and convalescent SARS-CoV-2 donors, cross-reactive memory B cells were present in pre-pandemic samples, whereas cross-reactive antibodies were barely detectable (203). Another study comparing the humoral response of children and adults in pre-pandemic era and SARS-CoV-2 convalescent sera showed that up to 40% of children had cross-reactive Abs, and their levels correlated with anti-HCoVs (204). Additionally, a notable increase of anti-HCoVs antibodies in convalescent sera was observed, suggesting a potential activation of cross-reactive memory B cells (204). The big proportion of children bearing anti-HCoVs antibodies underlies the higher proportion of infections with HCoVs compared to adults and may be a partial explanation behind the lower likelihood of children to develop severe COVID-19 (205).

However, there are conflicting reports on whether such cross-reactivity gets translated into a protective immunity, thus leading to less severe disease. Indeed, it has been shown that cross-reactive anti-HCoVs antibodies do not confer protection against SARS-CoV-2 infection nor to hospitalizations, though they are boosted upon SARS-CoV-2 infection (206, 207). On the contrary, another study showed that recent HCoVs infections are associated with less severe COVID-19 (208).

## 4 The Role of Microbiota

### 4.1 Upper Respiratory Tract Microbiota

The respiratory tract is the site of initial SARS-CoV-2 infection, which occurs in the context of a local microbiota that has been described to have a role in infection susceptibility and disease severity. Distinct alterations of the upper respiratory tract (URT) microbiota have been reported in SARS-CoV-2 infection: reduced microbial diversity (135, 209–215); depletion of commensal bacteria capable of controlling the constitutive production of type I and type III interferons (e.g. *Corynebacterium, Streptococcus, Dolosigranulum, Fusobacterium periodonticum* (135, 211, 214, 216)); increase of potential pathogen microbes (e.g. *Pseudomonaceae, Salmonella, Serratia, Haemophilus influenzae, Moraxella catarrhalis, Prevotella, Veillonella, Staphylococcus, Peptostreptococcus, Clostridium*) (135, 209–212). Such perturbations have been shown to be more pronounced in severely ill patients (135, 209, 211–213, 215, 216). As previous studies reported a role of *Fusobacterium periodonticum* in the metabolism of sialic acids (217), which can facilitate binding and viral entry (218, 219), depletion of *Fusobacterium periodonticum* in the upper respiratory tract microbiota may increase susceptibility to SARS-CoV-2 infection (216). Furthermore, reduction of microbial diversity, as well as depletion of *Corynebacterium* in nasopharyngeal microbiota, have been associated with lower levels of mucosal cytokines (IL-33, IFN-λ3, and IFN-γ) that may be important for viral control, and heightened levels of the chemokine CCL2, which has a role in recruiting pro-inflammatory monocytes into infected tissues (135). Similarly, enrichment of *Staphylococcus* in URT microbiota is positively correlated with nasopharyngeal viral load and levels of systemic inflammatory cytokines (IL-6 and TNF-α) (135).

Taken together, these observations suggest that disruption of the upper airway’s microbial homeostasis features COVID-19 in a severity-dependent fashion and is associated with dysregulation of local and systemic immune responses.

### 4.2 Lung Dysbiosis

Although classically assumed to be sterile, lungs harbor their own microbiota, albeit consisting of less abundant microbial populations as compared to gut microbiota (220). Due to the technical difficulties in low airways sampling, only a few studies have examined the lower respiratory tract microbiota in SARS-CoV-2 infection, hindering the possibility to draw firm conclusions. An observational study evaluating the lung microbiota in post-mortem lung biopsies from 20 fatal cases of COVID-19 showed that lung microbiota in these deceased patients was dominated by *Acinetobacter, Chryseobacterium, Burkholderia, Brevundimonas, Sphingobium*, and Enterobacteriaceae; besides, the lung fungal microbiota was dominated by *Criptococcus* (221). Another study displayed that lung microbiota of mechanically ventilated COVID-19 patients is enriched with oral commensals, such as *Mycoplasma salivarium, Prevotella oris*, and *Candida albicans*; further, deceased patients had higher total bacterial loads in their bronchoalveolar lavage (BAL) and a striking enrichment with *Mycoplasma salivarium* than patients who survived (222). As it has been previously shown that enrichment of the lower airway microbiota with oral commensals associate with a pro-inflammatory state in several diseases (223, 224), it is plausible that enrichment of lung microbiota with *Mycoplasma salivarium* in COVID-19 patients contributes to fueling lung and systemic inflammation, thus entailing a worse clinical outcome. A further study reported that lung microbiome of intubated COVID-19 patients is characterized by a low diversity of microbial communities and enriched with common respiratory pathogens (*Staphylococcus, Stenotrophomonas*), oral commensals (*Corynebacterium, Prevotella*) or gut-derived microbes (*Enterococcus* and Enterobacteriaceae such as *Escherichia* and *Klebsiella*) (215). Finally, a study based on BAL single-cell transcriptomic of COVID-19 patients in an intensive care unit (ICU) showed lung microbiota to be enriched with oral bacterial commensals, including *Mycoplasma salivarium* and *Prevotella* spp., in physical association with immune cells (i.e. following infection or internalization), which show high levels of inflammatory markers, suggesting a role of such bacteria in directly contributing to lung inflammatory response in severe COVID-19 (225). Thus, the enrichment of the lung microbiota with oral commensals in critically ill COVID-19 patients, which could be mainly explained by mechanical ventilation-associated aspiration, may also exacerbate inflammation in SARS-CoV-2 infection, as reported in other infectious and non-infectious diseases (223, 224).

### 4.3 Gut Barrier Dysfunction and Microbial Translocation

Disruption of the gut mucosal integrity with subsequent microbial translocation, i.e. leakage of intestinal microbes (bacteria and fungi) as well as their products from the gut to the systemic circulation, has been described in diverse infectious and non-infectious diseases as a factor fuelling systemic inflammation (226–228).

Severe COVID-19 patients presented high plasma levels of zonulin (229, 230), a mediator of tight junctions permeability that regulate the intestinal epithelial paracellular pathway inducing the disassembly of the protein ZO-1 from the tight junctional complex (227, 231, 232), and occludin (229), a tight junction structural protein. However, levels of intestinal fatty-acid binding protein (I-FABP), a marker of enterocyte apoptosis, are not heightened (229, 233, 234), suggesting that the alteration of the gut barrier in severe COVID-19 is due to an increased tight junctions’ permeability rather than to enterocyte death. Disruption in several intestinal metabolic pathways has also been displayed: plasma citrulline levels, an established marker of gut function (235), are lower in severe COVID-19 patients as compared to mild ones and controls (229), pointing to enterocyte dysfunction as an additional mechanism of gut leakage in these subjects.

Individuals with severe SARS-CoV-2 infection also show high circulating levels of lipopolysaccharide (LPS) (230), LPS binding protein (LBP) (229, 233, 234, 236), and β-glucan (229),as well as lower levels of EndoCAb-IgM (neutralizing antibodies against LPS endotoxin core antigen) (233), suggesting bacterial and fungal translocation in the systemic circulation. Bacterial proteins belonging to pro-inflammatory bacteria enriched in the gut microbiome of COVID-19 patients, such as *Burkholderia* spp.*, Pseudomonas* spp.*, Bifidobacterium longum*, have also been detected in blood samples of these subjects (236). Likewise, markers of microbial-mediated myeloid inflammation, i.e. sCD14, sCD163 (monocyte inflammation markers), and MPO (myeloperoxidase; neutrophil inflammation marker) are heightened in severe COVID-19 (229). Furthermore, plasma LPS positively correlates with zonulin (230), advocating that microbial translocation in COVID-19 is a direct consequence of gut barrier disruption.

The abovementioned markers of intestinal barrier permeability and microbial translocation have also been shown to correlate with high levels of several systemic inflammation and immune activation markers, pointing to a role of gut mucosal integrity disruption and microbial translocation in exacerbating systemic inflammation and thus contributing to disease severity (229, 236).

Interestingly, detectable plasma LPS, i.e. endotoxemia, has also been demonstrated to be associated with intra-hospital thrombotic complications in COVID-19 patients (230), suggesting that LPS may promote a prothrombotic state, further supported by the observation of higher levels of TIRAP phosphorylation in platelets (230), pointing to a TLR4-mediated activation by LPS (237). Additionally, plasma levels of LBP have been found heightened throughout the hospital stay in COVID-19 patients with elevated markers of cardiac involvement (N-terminal pro-B-type natriuretic peptide, troponin T, troponin I) and positively correlated to levels of inflammasome activity markers, which are associated with cardiac involvement too, suggesting that gut leakage with subsequent LPS-driven priming of the NLRP3 inflammasome may have a role in the pathogenesis of cardiac involvement during COVID-19 (234).

Intestinal barrier disruption during SARS-CoV-2 infection might be explained by several mechanisms: a direct viral infection of intestinal epithelial cells (238); down-regulation of ACE2 after viral infection of enterocytes (239); systemic inflammation sustained by cytokine storm (226); IL-6-mediated vascular damage (240); intestinal inflammation sustained by gut homing of T cells, as suggested by high plasma levels of CCL25, a gut homing marker, ligand for the chemokine receptor CCR9 (234, 241), and gut dysbiosis with subsequent mucosal inflammation (226, 242). The resulting increase in gut barrier permeability facilitates the passage of microbes and microbial products from the gut to the bloodstream, thus boosting systemic inflammation, which in turn promotes further gut leakiness, fostering a pro-inflammatory vicious cycle that can ultimately contribute to COVID-19 severity. Interestingly, well-established risk factors for severe COVID-19, like obesity and diabetes, are known to be associated with impairment of the gut barrier function and disturbances of the intestinal microbiota (243–245); hence, such alterations might contribute, at least in part, to the severity of disease in these subjects.

### 4.4 Gut Dysbiosis

As mentioned above, alteration of the gut microbiota, i.e. gut dysbiosis, is one of the mechanisms that can contribute to the intestinal barrier dysfunction observed in severe COVID-19 (242). Several alterations of the gut microbiota have been reported in SARS-CoV-2 infection: decreased microbial diversity (242, 246–248); decreased Firmicutes/Bacteroidetes ratio (242); depletion of beneficial butyrate-producing bacteria with anti-inflammatory and immunomodulatory potential (e.g. *Ruminococcaceae, Lachnospiraceae, Fusicatenibacter, Anaerostipes, Agathobacter, Eubacterium hallii, Clostridium butyricum, Clostridium leptum, Eubacterium rectale, Bifidobacterium bifidum, Bifidobacterium adolescentis, Bifidobacterium pseudocatenulatum, Alistipes onderdonkii*) (242, 246, 248–251); enrichment of potential pathogen microbes (e.g. *Streptococcus*, *Rothia, Veillonella, Erysipelatoclostridium, Actinomyces, Enterococcus*, Enterobacteriaceae*, Clostridium ramosum, Clostridium hathewayi, Clostridium innocuum, Coprobacilllus, Bacteroides nordii, Burkholderia contaminans, Bifidobacterium longum, Blautia*) (236, 242, 246, 248–251); overgrowth of opportunistic fungal pathogens (e.g. *Candida albicans, Candida auris, Aspergillus flavus, Aspergillus niger*) (252); skewing of microbial metabolic pathways (i.e. enhancement of glycolysis, fermentation, methionine biosynthesis, vitamin B12 biosynthesis, teichoic acid biosynthesis, and tryptophane catabolism) (229, 236).

It has been hypothesized that down-regulation of ACE2 on SARS-CoV-2-infected enterocytes could be the link between COVID-19 and gut dysbiosis (239). Indeed, it is known that ACE2 down-regulation in small intestine epithelial cells reduces the expression of the sodium-dependent neutral amino acid transporter B (0)AT1 (253), which in turn disturbs tryptophan absorption. As a result, the deficiency of tryptophan and its metabolite nicotinamide decreases the activity of the mTOR pathway, which regulates the expression of antimicrobial peptides in the intestinal mucosa, with subsequent alteration of the gut microbiota ecology (254).

Of note, most of these gut microbiota perturbations were found to be associated with higher levels of inflammatory and pro-thrombotic markers as well as more pronounced in severe and critical COVID-19 patients, suggesting that gut dysbiosis is involved in the magnitude of COVID-19 severity possibly *via* modulation of gut barrier permeability as well as host inflammatory and immune responses (229, 236, 242, 247–251).

### 4.5 Gut-Lung Axis

Although gut and lungs are anatomically distinct, complex pathways involving their microbial communities, i.e. microbiota, and immune cells, configure bidirectional interactions between intestinal and respiratory mucosa, known as gut-lung axis (255), which are believed to be involved in several physiologic and pathologic conditions (256, 257). Gut-lung axis perturbations involving gut barrier dysfunction with microbial translocation, gut dysbiosis, and lung dysbiosis, have also been implicated in COVID-19 immunopathogenesis and severity of disease (258).

The observation that the lung microbiota of SARS-CoV-2-infected patients can be enriched with bacteria that are typical of the gut microbiota, along with the evidence of gut barrier dysfunction and microbial translocation, suggests that translocation of microbes from the gut to the lungs *via* the bloodstream may occur in COVID-19, as previously described in mouse models of sepsis and in humans with ARDS (259), pointing to a role of the gut-lung axis in fueling lung/systemic inflammation and, in turn, disease severity.

## 5 Conclusions

In COVID-19 the majority of individuals do not have significant complications suggesting that there is a series of favorable events that successfully clear the virus. The factors that dictate whether a patient will develop the severe/critical form are still a matter of scientific debate. The comparison of pauci-symptomatic/asymptomatic individuals with symptomatic patients would be extremely helpful to define these factors. However, few studies managed to include a group of pauci-symptomatic/asymptomatic patients, thus limiting comparative assessments versus symptomatic and severe phenotypes. Additionally, heterogeneity in the definition of disease severity as well as in measurements of immune response further increase variability among studies. What is more, whereas at the beginning of the pandemic, the entire population was naïve to SARS-CoV-2 infection, i.e. no underlying immunity, two years after the beginning of the pandemic, the majority of subjects are now experienced to SARS-CoV-2 through either natural infection, vaccination or both, hence further hindering the identification of correlates of protection. Undoubtfully, while the activation and recruitment of the immune cells mentioned in the present article are initially part of the defensive response against SARS-CoV-2 infection, there seems to be a specific turning point in the natural course of the infection, in which the same immune pathways take a pathogenetic flavor, which overrides any later immune attempt to control the infection. The common thread towards a deleterious response appears to be the excessive activation, the skewing towards “non-useful” immune phenotypes, as well as exhaustion, altogether contributing to the pathological inflammatory and fibrotic process in severe COVID-19, eventually leading to ARDS and post-COVID fibrotic pulmonary sequelae. In the backdrop of a broad knowledge of the immune features associated with COVID-19 severity, very few underlying mechanisms have been identified so far. The identification of the factors underlying immune dysregulation in severe COVID-19 is crucial to understanding COVID-19 pathogenesis, defining early correlates of protection, and informing strategies of targeted therapeutic interventions.

## Author Contributions

RR: contributed to paper conceptualization, structured and wrote the review, created figures; MA: wrote the manuscript and created figures; AB-H: contributed to literature search and manuscript writing; VB: contributed to literature search and manuscript writing and figure design; ADM: edited the manuscript; GM: paper conceptualization and writing and revision of manuscript. All authors contributed to the article and approved the submitted version.

## Funding

This work was supported by grants from Fondazione Cariplo in collaboration with Regione Lombardia and Fondazione Umberto Veronesi (Rif. 2020-1355 and 2020-1376) and Fondazione di Comunità Milano.

## Conflict of Interest

The authors declare that the research was conducted in the absence of any commercial or financial relationships that could be construed as a potential conflict of interest.

## Publisher’s Note

All claims expressed in this article are solely those of the authors and do not necessarily represent those of their affiliated organizations, or those of the publisher, the editors and the reviewers. Any product that may be evaluated in this article, or claim that may be made by its manufacturer, is not guaranteed or endorsed by the publisher.
